# Period Estimation of Spread Spectrum Codes Based on ResNet

**DOI:** 10.3390/s23157002

**Published:** 2023-08-07

**Authors:** Han-Qing Gu, Xia-Xia Liu, Lu Xu, Yi-Jia Zhang, Zhe-Ming Lu

**Affiliations:** 1 School of Information Science and Engineering, Zhejiang Sci-Tech University, Hangzhou 310018, China; hanqinggu@163.com (H.-Q.G.); xlhit@126.com (X.-X.L.); xulu@zstu.edu.cn (L.X.); 2 School of Aeronautics and Astronautics, Zhejiang University, Hangzhou 310027, China

**Keywords:** direct sequence spread spectrum (DSSS), convolutional neural network (CNN), deep learning, spread spectrum code period estimation, ResNet

## Abstract

In order to more effectively monitor and interfere with enemy signals, it is particularly important to accurately and efficiently identify the intercepted signals and estimate their parameters in the increasingly complex electromagnetic environment. Therefore, in non-cooperative situations, it is of great practical significance to study how to accurately detect direct sequence spread spectrum (DSSS) signals in real time and estimate their parameters. The traditional time-delay correlation algorithm encounters the challenges such as peak energy leakage and false peak interference. As an alternative, this paper introduces a Pseudo-Noise (PN) code period estimation method utilizing a one-dimensional (1D) convolutional neural network based on the residual network (CNN-ResNet). This method transforms the problem of spread spectrum code period estimation into a multi-classification problem of spread spectrum code length estimation. Firstly, the In-phase/Quadrature(I/Q) two-way of the received DSSS signals is directly input into the CNN-ResNet model, which will automatically learn the characteristics of the DSSS signal with different PN code lengths and then estimate the PN code length. Simulation experiments are conducted using a data set with DSSS signals ranging from −20 to 10 dB in terms of signal-to-noise ratios (SNRs). Upon training and verifying the model using BPSK modulation, it is then put to the test with QPSK-modulated signals, and the estimation performance was analyzed through metrics such as loss function, accuracy rate, recall rate, and confusion matrix. The results demonstrate that the 1D CNN-ResNet proposed in this paper is capable of effectively estimating the PN code period of the non-cooperative DSSS signal, exhibiting robust generalization abilities.

## 1. Introduction

A direct sequence spread spectrum signal has good anti-interference performance, low interception probability and other excellent characteristics, and it is widely used in military and civil communication systems such as identification of friend or foe, satellite communication, indoor wireless communication, WIFI, etc. However, these characteristics will also make signal detection and parameter estimation more difficult in non-cooperative situations. The estimation of the spreading code period is fundamental to the estimation of other parameters in non-cooperative DSSS signal processing. The period estimation of spread spectrum codes will be explored in this paper.

With the continuous development of signal detection technology and parameter blind estimation technology, the methods for blind estimation of DSSS signal spread spectrum code (also known as PN code) period are also constantly improving. Currently, the mature PN (Pseudo-Noise, PN) code period estimation methods that have been studied mainly include the correlation method, cepstrum method, power spectrum method, power spectrum reprocessing method [1], and correlation entropy method [2]. Some researchers studied existing DSSS signal-processing techniques and proposed an autocorrelation estimator to estimate the PN code period. Dong and Hu [3] proposed a delay multiplication-based estimation method for the pseudo-random sequence estimation of long pseudo-random code direct spread spectrum signals, which can not only estimate the pseudo-random code sequence but also estimate the information code period and its synchronization information. Some researchers proposed an estimation method that combines delay multiplication with correlation spectrum analysis based on the periodicity of the peaks that appear after the delay multiplication of direct spread spectrum signals, and they achieved the estimation of a symbol period with direct spread spectrum signals. The cepstrum method mainly uses logarithmic operation to make the frequency domain spectral lines of the direct spread spectrum signal more obvious and easy to detect, thereby improving detection performance. Some researchers achieved the period detection of direct spread spectrum signals by using power spectrum averaging to reduce the impact of pulse noise on detection performance. Some researchers proposed a narrow window autocorrelation cepstrum estimation method based on the classical cepstrum method, and they proposed a new peak search strategy based on least squares fitting to address the case of peak misdetection, further improving estimation performance. Some researchers solved the problem of difficult estimation of the pseudo-code period of the direct spread spectrum signal in multipath environments and calculated the power spectrum of the DSSS signal once again. They found that the spike pulses at the integer multiple of the spreading code period in the secondary power spectrum were much more pronounced than those in the primary power spectrum. By detecting the distance between these spike pulses, the estimation of the pseudo-code period of the direct spread spectrum signal in multipath environments was achieved. Bai et al. proposed a period estimation method for power spectrum reprocessing to address the challenge of weak direct spread spectrum signal period estimation under strong narrowband interference [4]. This method first uses the overlapping window FFT algorithm to transform the received signal into the frequency domain for strong narrowband interference cancellation and then performs period estimation. On the basis of previous research, Yu et al. proposed an estimation method for power spectrum reprocessing based on wavelet decomposition [5], which improves the estimation performance in low signal-to-noise ratio situations. Sheng et al. [1] proposed an improved estimation method for power spectrum reprocessing to address issues such as peak energy leakage and false peak interference by modifying segment lengths, overlapping segments, and extracting non-adjacent peaks. Some researchers studied the relationship between the correlation entropy of direct spread spectrum signals and the period of pseudo-code, and they proposed an estimation method based on delay multiplied correlation entropy. Experiments have shown that the performance of this method is superior to cepstrum and correlation methods.

Deep learning is an important method of artificial intelligence technology, which has been applied in various fields such as image processing [6,7,8], speech recognition [9], emotion recognition [10], text processing [11], and social services [12,13,14,15]. In the field of signal processing, deep learning is mainly used in intelligent receivers [16], signal debugging method recognition [17,18], radar signal classification [19], image-based radio signal recognition [20], and other aspects. Chen et al. proposed a new deep learning framework for signals [21], which can directly input signals into the network without pre-extracting features. He et al. [22] proposed a Residual Network (ResNet) in 2016 to address the problem of gradient explosion after network model deepening, which can make the network model deeper. After the ResNet model was proposed, it has been widely applied in fields such as image recognition [23,24] and signal processing [25]. Jin and Kim proposed a PN code length estimation based on deep learning for the first time [26], using a basic CNN network to achieve PN code length estimation for direct spread spectrum signals with lengths of 16, 32, 64, and 128. However, this method has poor estimation performance in low signal-to-noise ratios and cannot achieve an estimation of other commonly used PN code lengths.

In this paper, we propose a PN code period estimation method using a 1D CNN-ResNet to address the issues of peak leakage and false peaks in traditional spread spectrum code period estimation methods such as the power spectrum method and cepstrum method under low signal-to-noise ratio conditions. The main idea of this method is to change the calculation of the spreading code period to the calculation of the spreading code length based on the fact that the spreading code period is equal to the spreading code length multiplied by the chip width. On this basis, the problem of estimating the spreading code period of DSSS signals modulated by commonly used spreading code lengths 127, 255, 511, 1023, and 2047 is modeled as a multi-class spreading code length problem, and the ResNet network is used to achieve the length estimation of the spreading code and then, based on their relationship, implement spread spectrum code period estimation. During the network training, directly using the I and Q channel data of these types of direct spread spectrum signals as dual-channel inputs to train the network enables distinguishing the spread spectrum code length of the input signal.

- Transforming the period estimation of the PN code into a multi-classification problem, as the length of the PN code can only be $2^{n}-1$, in this paper, and proposing a CNN-ResNet based model to estimate the DSSS signal on the basis of previous research.
- Generating the DSSS signals in a Gaussian channel with PN code periods of 127, 255, 511, 1023, and 2047, which serve as training and testing data sets. The model is then trained by BPSK-modulated signals.
- Analyzing the estimation performance of the model under different SNRs in a simulation experiment and studying the PN code period estimations at the SNR of −10 and −8 dB through the confusion matrix.
- Testing the trained model to examine the DSSS signal modulated by QPSK. The model can also effectively estimate the PN code period of the QPSK-DSSS signal. The result proves that the model in this paper has strong generalization of the period estimation of the spreading code.

The rest of this paper is structured as follows. Section 2 presents the signal model and proposes the method for the period estimation of PN codes based on the CNN-ResNet model. Section 3 details the simulation experiment and result analysis, and Section 4 concludes the content of the paper and discusses future work directions.

## 2. Proposed Methodology

### 2.1. Overall Framework

**Figure 1:**
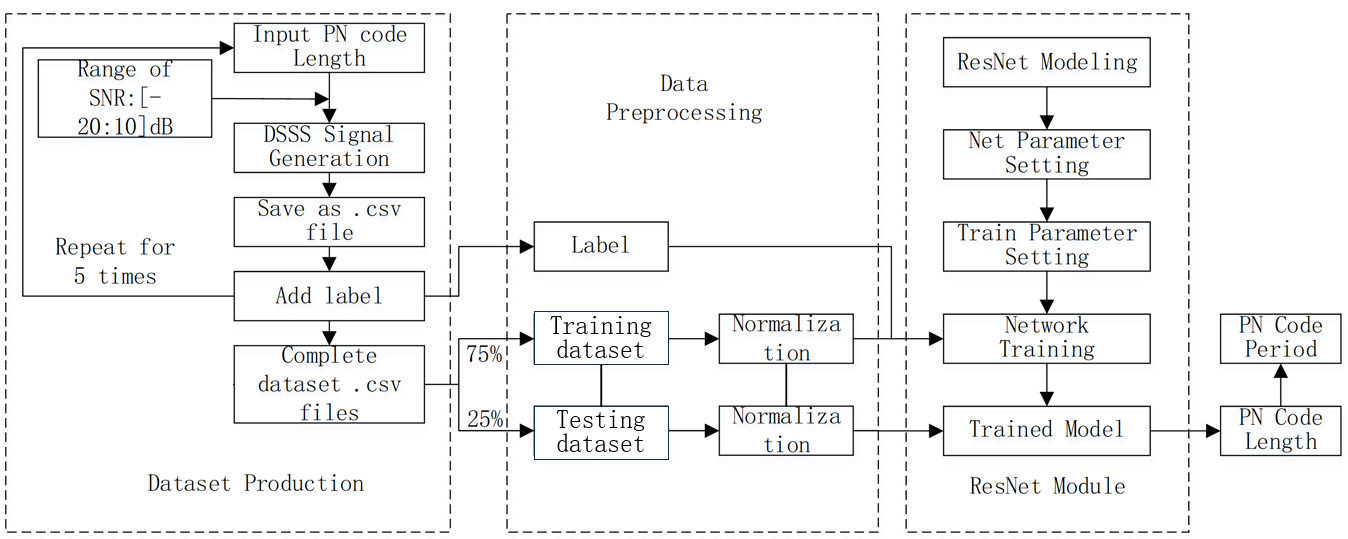
Overall framework diagram of PN code period estimation based on ResNet.

The classic algorithms of the period estimation of the spread spectrum code include the delay multiplication correlation method, cepstrum method, power spectrum method and its reprocessing method. This paper introduces deep learning into spread spectrum code period estimation and proposes a ResNet-based spread spectrum code period estimation method. Firstly, based on the fact that the period of the spread spectrum code is equal to the length of the spread spectrum code multiplied by the chip width, the problem of estimating the period of the spread spectrum code is first transformed into the problem of estimating the length of the spread spectrum code. Then, according to the characteristic that the length of the spread spectrum code can only be $2^{n}-1$, the direct-sequence spread spectrum signals modulated by the spread spectrum codes with different lengths are regarded as the same kind of signals, and then the problem of estimating the length of the spread spectrum code is transformed into a multi-classification problem, and the period of the spread spectrum code is estimated indirectly through the method of deep learning. Since the signal is one-dimensional, and CNN has the advantages of fewer trainable parameters, fast rate of convergence, parallel computing, etc., we choose to use the one-dimensional convolutional neural network based on the residual structure to complete the estimation of the spread spectrum code length of direct spread spectrum signals. The overall framework of PN code period estimation based on ResNet is shown in Figure 1. It is worth mentioning that the .csv format data set file in Figure 1 contains DSSS signals with different spreading code lengths and different signal-to-noise ratios.

### 2.2. ResNet Network Model

**Figure 2:**
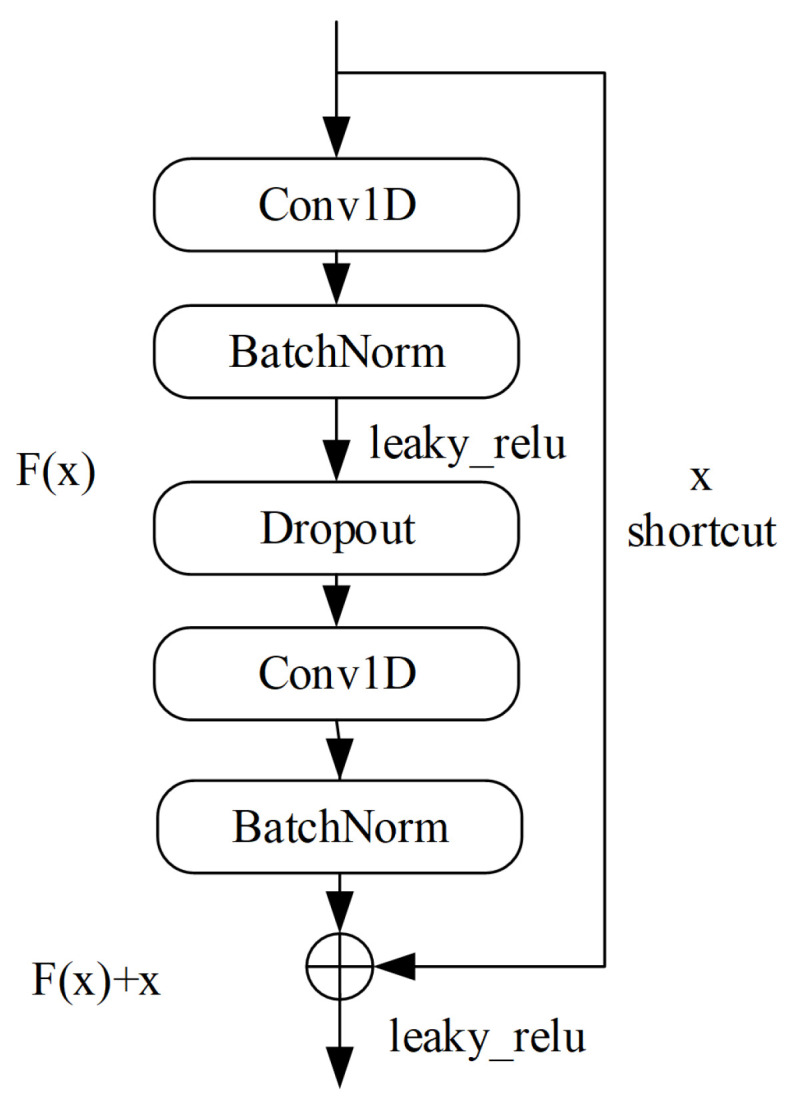
Structure of residual module in this paper.

Due to the fact that the signal dataset contains signals with low signal-to-noise ratios and the overall differences between signals are small, the requirement for network fitting ability is relatively high, and simple CNN networks cannot meet this requirement. Although deepening the number of layers in the network model can improve the network fitting ability, increasing the number of layers after the network model is deepened to a certain extent can lead to problems such as gradient disappearance or gradient explosion. To avoid this issue, we chose to use the ResNet residual network, use two one-dimensional convolutional layers to build the basic residual module, and add a dropout layer between the two convolutional layers to reduce overfitting, as shown in Figure 2. The activation function uses the Leaky_Relu function to replace the commonly used Relu function, because the Relu function will turn all negative values into 0, making the gradient of this neuron always 0, and neurons on the negative axis no longer learn, resulting in a decrease in the accuracy of the estimation results. Meanwhile, the Leaky_Relu function introduces a small constant on the basis of the Relu function, which can preserve some negative axis values, reduce the occurrence of silent neurons, and ensure that the negative axis information does not completely disappear, thereby improving the accuracy of the estimation results.

**Figure 3:**
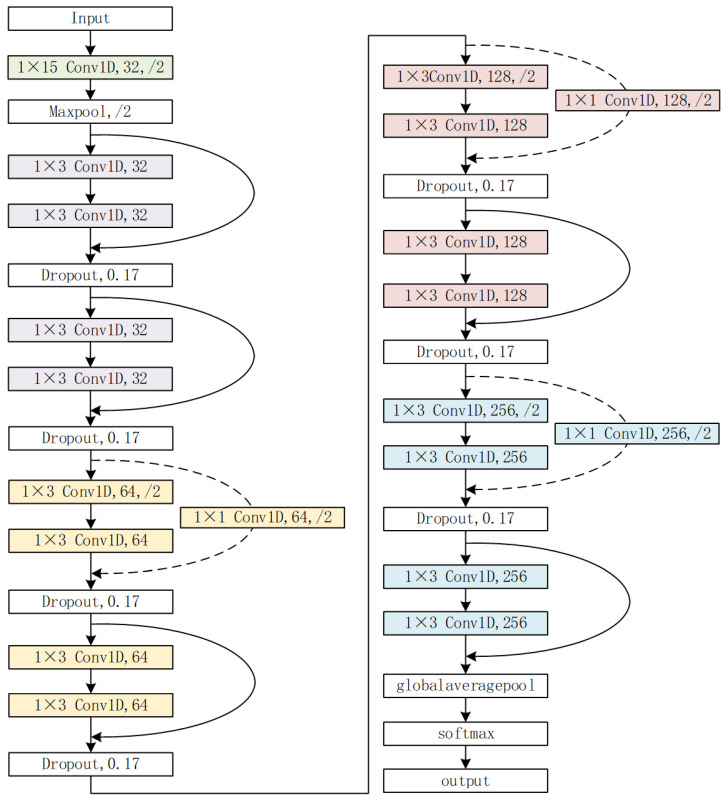
Network model framework diagram.

The overall structure of the network model designed in this article is composed of a single convolutional layer, a maximum pooling layer, eight basic residual modules, a global average pooling layer, and a softmax layer sequentially connected. The details and parameters of the model are shown in Figure 3 and Table 1. Each residual module contains two convolutional layers, two BatchNorm layers, and one dropout layer. In order to make the overall framework diagram of the network model clearer, the BatchNorm layer and dropout layer in each residual block are omitted from the overall network structure diagram. In the figure, “m×nConv1D,N,/M” indicates that the convolutional kernel size in this layer is m×n. The number of channels is *N*, the downsampling factor is *M*, Maxpool represents the one-dimensional maximum pooling layer, and Globalaveragepool represents the one-dimensional global average pooling layer. The additional “1 × 1” convolutional layer introduced in the residual section of Figure 3 is used to solve the problem of data dimension mismatch between the input and output of the residual block.

### 2.3. Network Model Evaluation Indicators

In the training process, TP represents the number of positive samples with correct classification, and the Accuracy and Loss functions are used to measure the quality of the model. Equation (1) represents the calculation method for accuracy, $TP_{i}$ represents the number of correct classifications for the *i*-th type, and *n* represents the total number of classifications. Cross-entropy is chosen as the loss function of the network model to measure the deviation between the real value and the predicted value. If p(x) is the true distribution of signal *x* and q(x) is the predicted distribution, the loss function based on cross-entropy is shown in Equation (2).

(1) $$Accuracy=\frac{\sum_{i=1}^{n}TP_{i}}{number\;of\;samples}$$

(2) $$Loss=-\sum_{x}p\left(x\right)\log q\left(x\right)$$

Recall is used to measure the quality of the model’s prediction results, as shown in Equation (3). TP represents the number of positive samples correctly classified, and FP represents the number of negative samples determined from the actual positive samples.

(3) $$Recall=\frac{TP}{FP+TP}$$

## 3. Simulation Experiment and Result Analysis

### 3.1. Dataset Production

First, the original information sequence is randomly generated; then, it is encoded with m-sequences of different lengths generated through the linear displacement register, and finally, the encoded signal is modulated with the BPSK modulation method to obtain the direct-sequence spread spectrum signals. The length of each signal is 8192, and the real and imaginary parts are the data of the I and Q channels. The I/Q channels are used as the channel dimensions of the dataset. Therefore, the size of a single sample in the dataset is 1×8192×2. The lengths of the m sequences used are 127, 255, 511, 1023, and 2047, respectively, with a signal-to-noise ratio range of [−20:2:10] dB. Under each signal-to-noise ratio, 200 sample signals of different spread spectrum code lengths are generated and combined together to form a complete dataset, allowing the trained individual network to adapt to different signal-to-noise ratios. Before training, the entire dataset needs to be unordered and then divided into training and testing sets in a 3:1 ratio.

### 3.2. Training Environment and Process

The model training is completed on a computer with a CPU model of Intel(R) Core(TM) i7-10875H @ 2.30 GHz (Intel, Santa Clara, CA, USA), with a running memory of 32 GB and a GPU model of NVIDIA GeForce RTX2060 (NVIDIA, Santa Clara, CA, USA). The network training parameters are shown in Table 2. There are two dropout parameters, with a dropout value of 0.15 between the two convolutional layers in each residual module and a dropout value of 0.17 between the different residual modules.

**Figure 4:**
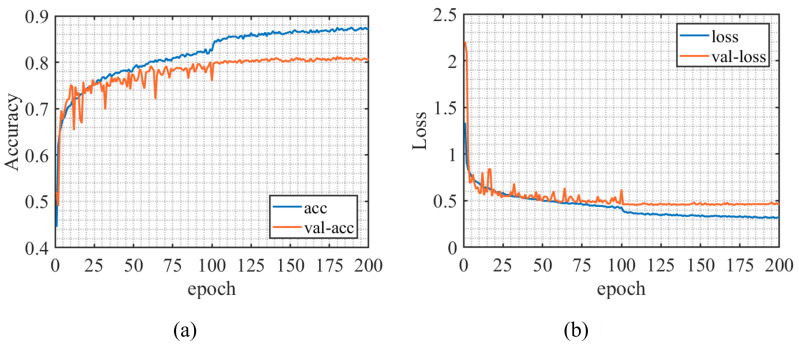
Training process. (**a**) Accuracy curve, (**b**) Loss function curve.

Figure 4a,b, respectively, show the change of the accuracy and loss function on the training set and test set during the training of the ResNet-based CNN network. It can be seen from Figure 4a that after training 23 epochs, the accuracy on the training set and the test set has reached 75%, which means the rate of convergence of the model is fast. After the final training, the model achieved an accuracy rate of 87.34% on the training set and 80.70% on the test set. Due to the fact that the signal-to-noise ratio of the dataset used for model training and testing during the training process ranges from −20 to 10 dB, the performance of the network model is relatively poor at low signal-to-noise ratios, resulting in a decrease in overall accuracy. The accuracy of the model under different signal-to-noise ratios was specifically demonstrated in subsequent experimental results analysis.

### 3.3. Estimation Performance Analysis under Different Signal-to-Noise Ratios

In order to verify the performance of the network model in estimating the spread spectrum code length of DSSS signals under different signal-to-noise ratios, the selected spread spectrum code lengths are 127, 255, 511, 1023 and 2047. The signal-to-noise ratio range is [−20, 0] dB; with each increase of 2 dB, the modulation method adopts BPSK modulation. A total of 100 direct spread spectrum signals with different spreading code lengths were generated for each signal-to-noise ratio.

**Figure 5:**
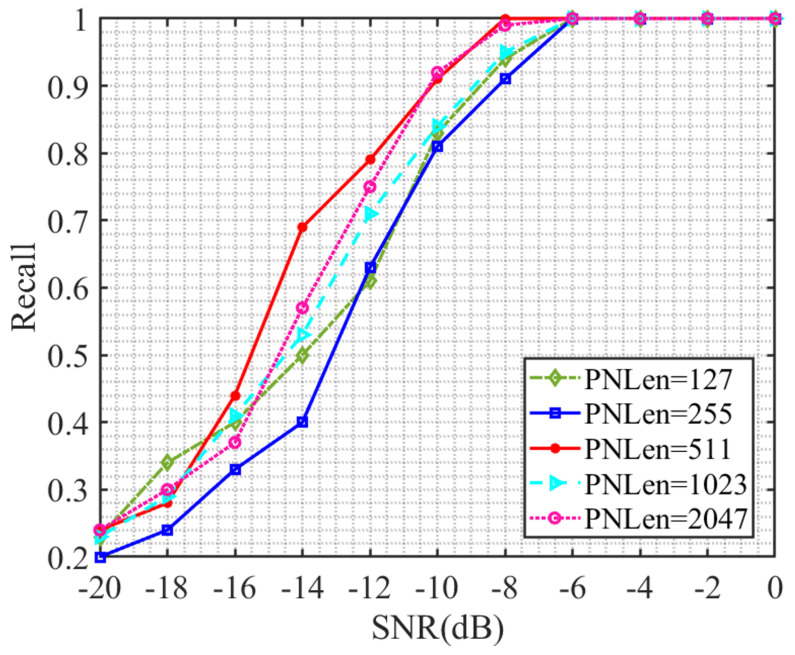
Recall rate versus signal-to-noise ratio curve.

Separate tests were conducted on direct spread spectrum signals with different spreading code lengths under different signal-to-noise ratios, and the test results are shown in Figure 5. From Figure 5, it can be seen that the estimated recall rate continues to increase as the signal-to-noise ratio increases. When the signal-to-noise ratio is higher than −13 dB, the recall rate of the estimated result reaches over 50%. When the signal-to-noise ratio reaches −8 dB or above, the recall rate of the estimated result reaches over 90%.

Due to the identical length of each direct spread spectrum signal, the longer the selected spread spectrum code, the shorter the length of the original information sequence contained in the spread spectrum signal. Observing the ranking of the recall rate from high to low corresponding to the length of the spread spectrum code at the same signal-to-noise ratio in Figure 5, it is found that this ranking result is not completely the same, and there is no obvious pattern. Therefore, it can be explained that the recall rate of estimating the length of the spread spectrum code using this model is independent of the length of the original information sequence contained in the direct spread spectrum signal. At the same time, it can be found in Figure 5 that at −14 dB and −12 dB, the recall rates of different spread spectrum code length estimates differ greatly. A specific analysis will be made through the confusion matrix in Section 3.5.

### 3.4. Estimation Performance Analysis under QPSK Modulation Mode

**Figure 6:**
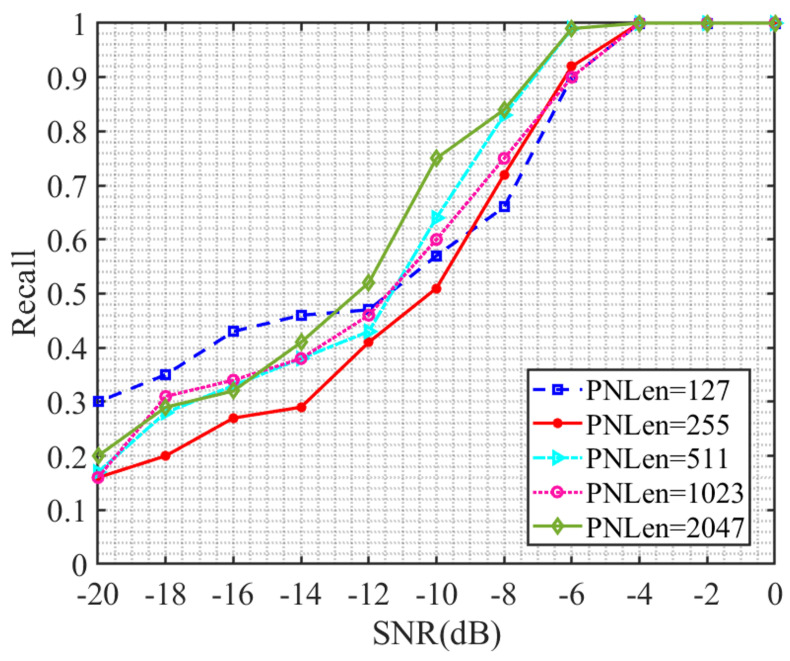
Recall rate versus signal-to-noise ratio curve.

The modulation method of the direct spread spectrum signal in the dataset used in the training of the network model in this article is BPSK. Here, the QPSK-modulated direct spread spectrum signal is used to verify the generalization ability of the network model. Figure 6 shows the change in recall rate of the direct spread spectrum signal modulated by QPSK in the signal-to-noise ratio range of −20 to 0 dB, using the model proposed in this paper for spreading code length estimation. The spread spectrum code lengths of the direct spread spectrum signals selected in the experiment are 127, 255, 511, 1023 and 2047; the signal-to-noise ratio increases by 2 dB each time.

It can be seen from Figure 6 that the network model is also suitable for QPSK DSSS signals. The recall rate of PN code length estimation for QPSK DSSS signals modulated with different lengths of PN codes steadily increases with the increase of signal-to-noise ratio. When the signal-to-noise ratio is higher than −6 dB, the recall rate of the estimated results of the model in this paper reaches over 90%. At −4 dB, the recall rate reaches 100%, which reduces the overall estimation performance of the model by 2 dB compared to the BPSK-DSSS signals.

By observing the ranking of the corresponding spread spectrum code length for each same signal-to-noise ratio recall rate from high to low, it is found that this ranking result is not completely the same and there is no obvious pattern. Therefore, similar to the previous section, the recall rate of the model’s estimation results remains unaffected by the length of the original information sequence contained in the signal under QPSK modulation.

### 3.5. Confusion Matrix Analysis

**Figure 7:**
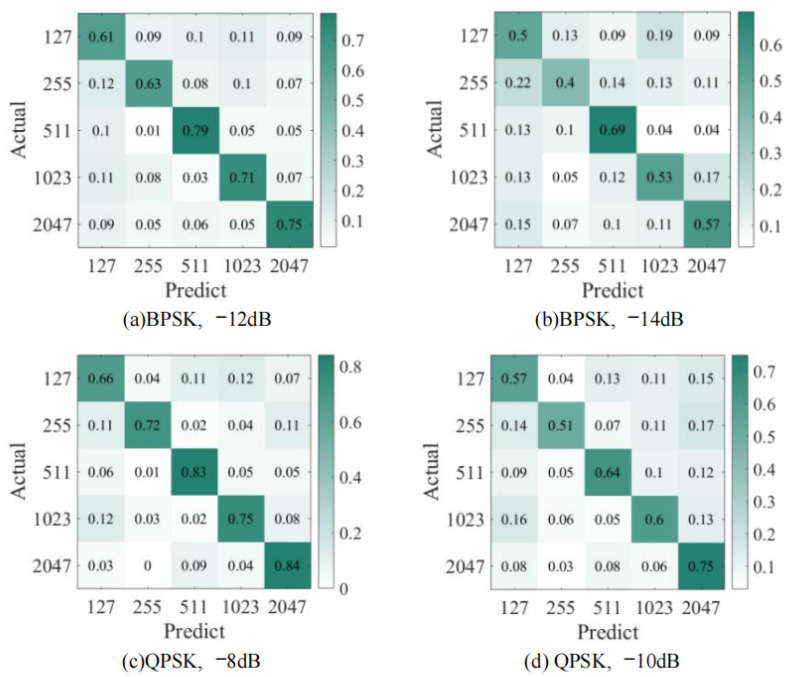
Confusion matrix. (**a**) Confusion matrix of BPSK-DSSS signals with different PN code lengths at −12 dB. (**b**) Confusion matrix of BPSK-DSSS signals with different PN code lengths at −14 dB. (**c**) Confusion matrix of QPSK-DSSS signals with different PN code lengths at −8 dB. (**d**) Confusion matrix of QPSK-DSSS signals with different PN code lengths at −10 dB.

Figure 7a,b show the confusion matrix of BPSK-modulated DSSS signals with different PN code lengths at −12 dB and −14 dB, Figure 7c,d show the confusion matrix of QPSK-modulated DSSS signals with different PN code lengths at −8 dB and −10 dB, and the diagonal value in the confusion matrix represents the recall rate. Observing the sum of two numbers with opposite row and column numbers in the first row and the first column of these four images in Figure 7, it can be found that the model in this paper is most likely to confuse the two cases with PN code lengths of 127 and 1023 in most cases, indicating that the network still needs further strengthening in extracting different features of these two signals. It can be found from the data of the diagonal of the Confusion matrix in Figure 7 that the recall rate of the estimated result of the spread spectrum code lengths of 127 and 255 is the lowest; this result illustrates that they are the most difficult to be accurately estimated. Observing Figure 7a,b, it can be observed that in the case of a low signal-to-noise ratio, direct spread spectrum signals with PN code lengths of 255, 511, 1023, and 2047 are most likely to be misjudged as PN code lengths of 127 when using BPSK modulation. At the same time, the probability of a signal with PN code lengths of 127 being misjudged as PN code lengths of 1023 increases the fastest as the signal-to-noise ratio decreases. By comparing Figure 7a–d, it can be observed that the total probability of DSSS signals with different PN code lengths being misjudged as adjacent PN code lengths increases as the signal-to-noise ratio decreases.

### 3.6. Comparison of Estimation Performance between Different Methods

**Figure 8:**
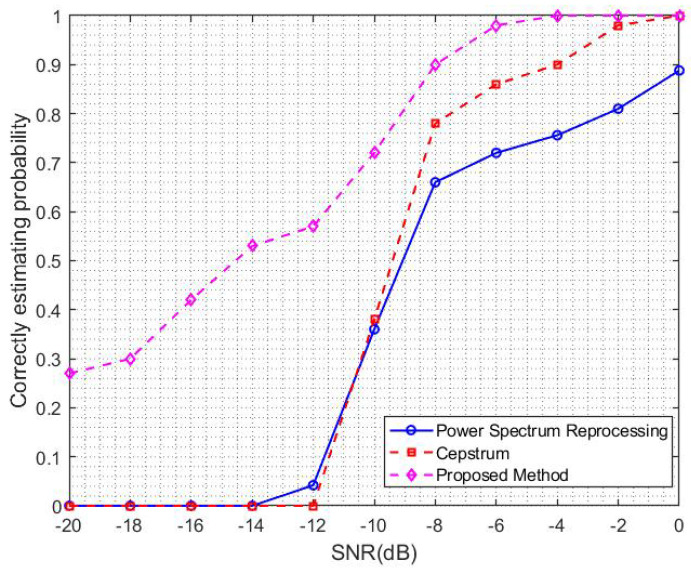
Comparison of spread spectrum code period estimation performance.

This section compares and analyzes the performance of the ResNet-based spread spectrum code period estimation method with the power spectrum reprocessing method and the cepstrum method in the traditional spread spectrum code period estimation methods through simulation experiments. The DSSS signal in this experiment contains a 65-bit information code with a length of 127 spreading codes. The spreading code sequence type is an m sequence with a modulation method of BPSK. The noise is Gaussian white noise, and the signal-to-noise ratio range is [−20, 0] dB. The two methods perform 100 simulated reality tests under a single signal-to-noise ratio, and the information codes in each experiment are randomly generated. If there is a relative error, the estimation result is considered correct. The curve of the probability of correctly estimating the period of the spread spectrum code with respect to the signal-to-noise ratio obtained through simulation experiments is shown in Figure 8.

From Figure 8, it can be seen that the correct estimation probability of the spread spectrum code period by all three methods increases continuously with the increase of signal-to-noise ratio. At the same time, it can be found that the power spectrum reprocessing method and the cepstrum method have completely failed below −14 dB and cannot estimate the correct spread spectrum code period, while the method proposed in this paper still has a probability of 0.50 to estimate the correct spread spectrum code period. At −6 dB, the correct estimation probability of the proposed method reaches 1, while the correct estimation probability of the power spectrum reprocessing method and the cepstrum method are only 0.72 and 0.86, respectively, which are 0.28 and 0.14 away from the proposed method. The correct estimation probability of the power spectrum reprocessing method reaches 0.88 at 0 dB, while the correct estimation probability of the proposed method has already reached 0.88 at −9 dB. Therefore, the overall estimation performance of the proposed method is improved by 9 dB compared to the power spectrum reprocessing method. Similarly, our method improves the estimation performance by 4 dB compared to the cepstrum method.

### 3.7. Discussion

With a vision toward exploring new frontiers in the estimation of the PN code period, this paper transformed it into a classification problem of DSSS signals with different spreading code periods, leveraging the power of a 1D CNN-ResNet. Upon training and verifying the model using BPSK modulation, the model was then put to the test with QPSK-modulated signals, and the estimation performance was analyzed through metrics such as loss function, precision rate, recall rate, and confusion matrix.

The model achieved an accuracy rate of 87.34% on the training set and 80.70% on the test set. Due to the fact that the signal-to-noise ratio of the dataset used for model training and testing during the training process ranges from −20 to 10 dB, the performance of the network model is relatively poor at low signal-to-noise ratios, resulting in a decrease in overall accuracy. In addition, we designed a comparative experiment between the proposed algorithm, the power spectrum reprocessing algorithm and the cepstrum algorithm, and the results showed that the overall estimation performance of our method was improved by 9 dB and 4 dB compared to the power spectrum reprocessing method and the cepstrum method. Although the model was proved to be effective, as the overall performance of QPSK and BPSK-modulated signals differs by 2 dB, there remains room for improvement. Furthermore, it is regrettable that the data used in the above studies were generated through simulation and we did not use actual spread spectrum signals for experiments; thus, the practicality of the proposed method cannot be confirmed yet.

## 4. Conclusions

In the realm of blind parameter estimation of non-cooperative DSSS signals, PN code period estimation is a critical aspect. The traditional time-delay correlation algorithm encounters the challenges such as peak energy leakage and false peak interference. As an alternative, we introduce a PN code period estimation method utilizing a one-dimensional (1D) convolutional neural network based on the residual network (CNN-ResNet). According to the relationship between the period and the length of the PN code, we transform the problem of estimating the period of the PN code into the problem of estimating the length of the PN code. Then, the PN code length estimation is transformed into a multi-classification problem. The training dataset includes DSSS signals modulated by BPSK with different spread spectrum code lengths with signal-to-noise ratios ranging from −20 to 10 dB. The experimental results show that the accuracy of the estimation results has reached 100% when the model is at −6 dB. At the same time, the estimation performance under QPSK modulation was also verified. The experimental results showed that the model in this paper is still suitable for the period estimation of QPSK-modulated DSSS signals, but the overall performance decreased by 2 dB. As a result, we will set our sights on investigating the ways to boost the model’s accuracy and generalization. In the future, we will try to use LSTM to complete the PN code period estimation of DSSS signals. Moreover, testing the actual DSSS signal also constitutes part of our future work.

## Figures and Tables

**Table 1:** Network model parameters.

Network Layer Name	Output Dimension	Network Layer Name	Output Dimension
Input	1×8192×2	Dropout	1×1024×64
Conv1D	1×4096×32	Residual-block 5	1×512×128
Maxpool	1×2048×32	Dropout	1×512×128
Residual-block1	1×2048×32	Residual-block 6	1×512×128
Dropout	1×2048×32	Dropout	1×512×128
Residual-block 2	1×2048×32	Residual-block 7	1×256×256
Dropout	1×2048×32	Dropout	1×256×256
Residual-block 3	1×1024×64	Residual-block 8	1×256×256
Dropout	1×1024×64	Globalaveragepool	1×1×256
Residual-block 4	1×1024×64	Softmax	1×1×5

**Table 2:** Model training parameters.

Parameter	Parameter Value
Initial learning rate	0.001
Training rounds	200
Small batch size	8
Learning rate decline cycle	100
Learning rate decline coefficient	0.1
Droupout Rate	0.15/0.17
Optimizer	Adam

## Data Availability

The study did not report any data.
